# Case Report: Metagenomic Next-Generation Sequencing in Diagnosis of Talaromycosis of an Immunocompetent Patient

**DOI:** 10.3389/fmed.2021.656194

**Published:** 2021-03-29

**Authors:** Jiejun Shi, Naibin Yang, Guoqing Qian

**Affiliations:** Department of Infectious Diseases, Ningbo First Hospital, Ningbo University, Ningbo, China

**Keywords:** talaromyces marneffei, immunocompetent, next-generation sequencing, anti-fungal treatment, case report

## Abstract

**Background:** Talaromycosis is a serious fungal infection which is rare in immunocompetent people. Since its clinical manifestations lack specificity, it is easy to escape diagnosis or be misdiagnosed leading to high mortality and poor prognosis. It is necessary to be alert to the disease when broad-spectrum antibiotics do not work well in immunocompetent patients.

**Case Presentation:** A 79-year-old man was admitted to our Infectious Diseases Department for recurrent fever and cough. Before admission he has been treated with piperacillin-tazobactam, moxifloxacin followed by antituberculous agents in other hospitals while his symptoms were not thoroughly eased. During the first hospitalization in another hospital, he has been ordered a series of examination including radionuclide whole body bone imaging, transbronchial needle aspiration for subcarinal nodes. However, the results were negative showing no neoplasm. After being admitted to our hospital, he underwent various routine examinations. The initial diagnosis was bacterial pneumonia, and he was given meropenem injection and tigecycline injection successively, but there were no improvement of symptoms and inflammatory indicators. In the end, the main pathogen *Talaromyces marneffei* was confirmed using Metagenomic Next-Generation Sequencing (mNGS), and his clinical symptoms gradually relieved after targeted antifungal treatment using voriconazole.

**Conclusion:** When empirical anti-infective treatment is ineffective, it is necessary to consider the possibility of opportunistic fungal infections on immunocompetent patients. mNGS, as a new generation of pathogenic testing methods, can often detect pathogenic bacteria faster than traditional methods, providing important help for clinical decision-making.

## Introduction

Talaromycosis is an opportunistic fungal infection caused by *Talaromyces marneffei* (*T. marneffei*), the only thermal dimorphic fungus of Penicillium, which is endemic to Southeast Asia but rare in the Ningbo city of Zhejiang province. Excrement of Rhizomys that were carrying *T. marneffei* may contaminates surroundings, leading to infections. Susceptible people may be infected through alimentary tract, respiratory tract and broken skin or mucosa contact (1). Since the clinical manifestations lack specificity, it is easy to escape diagnosis and be misdiagnosed especially amongst immunocompetent patients. It is one of the severe opportunistic infections in HIV patients. Recently a small but increasingly number of HIV-negative patients with abnormal immunity, such as patients of connective tissue disease, organ transplant recipients, and long-term users of immunosuppressive agents, were reported to be infected. It rarely infects immunocompetent people, though when it happens the mortality were higher, maybe due to delayed diagnosis (2, 3). Here we presented an immunocompetent Ningbo native suffered from *T. marneffei* which was hard to identify by conventional detection methods. Finally, the fungus was confirmed by mNGS and prolonged sputum culture.

## Case Report

**Figure 1:**
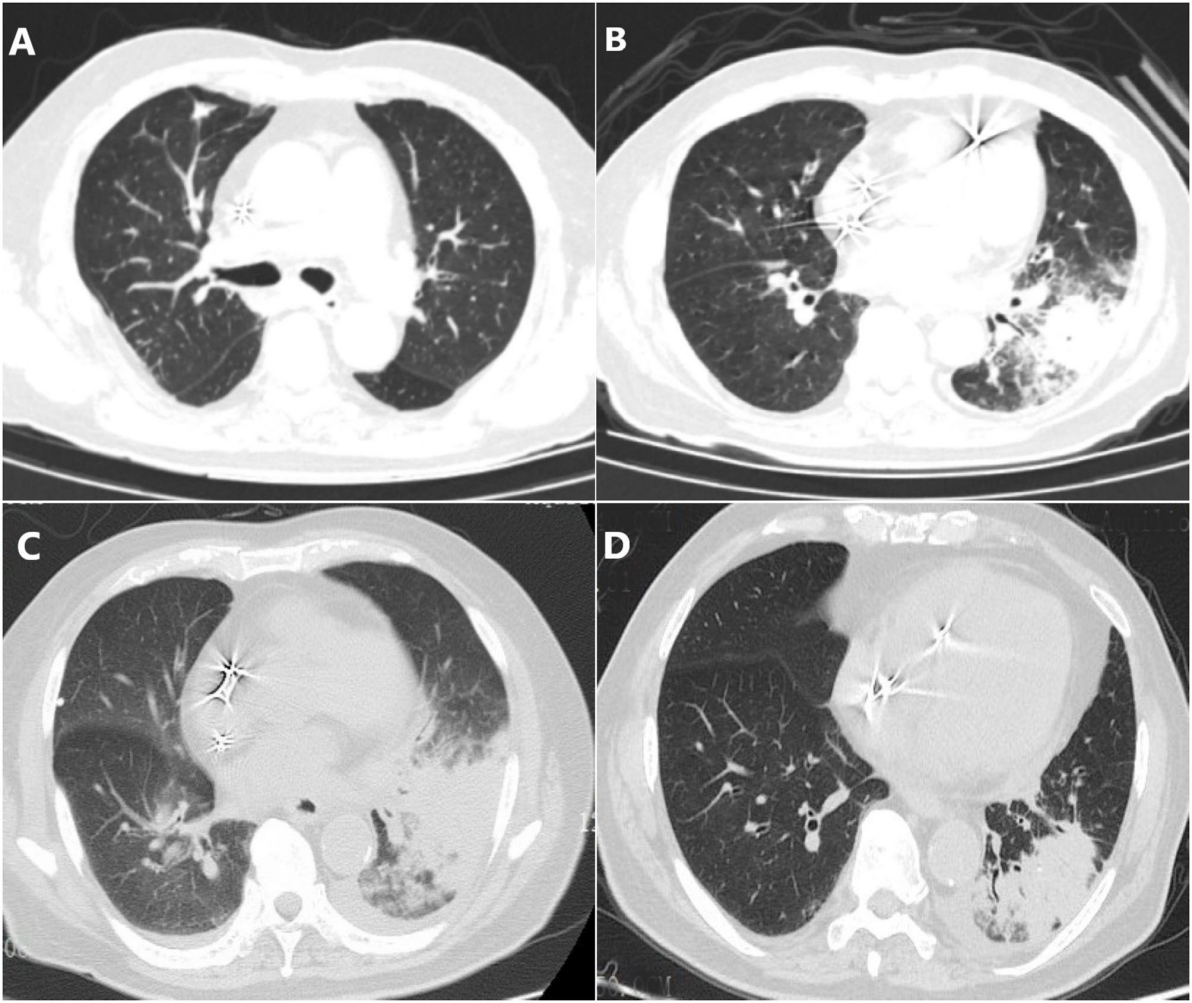
Chest CT scans in different phases. **(A)** Ground-glass nodules in the upper lobe of the right lung with multiple enlarged lymph nodes in the mediastinum which were suspected of tumors. **(B)** Infectious lesions in both lungs, left hilar shadow increased, and mediastinal lymph nodes slightly enlarged. **(C)** The area of pneumonia was larger than before and pleural fluid appeared. **(D)** Pneumonia and pleural effusion were both absorbed obviously.

A 79-year-old man was presented to our hospital with 1-month cough and fever. He has visited two other local hospitals for more than three times where a series of examinations were performed. His chest computerized tomography (CT) scan revealed ground-glass nodules in the upper lobe of the right lung with multiple enlarged lymph nodes in the mediastinum which were suspected of tumors (Figure 1A). In order to clarify the nature of the lesion he further took a needle biopsy of lymph nodes under bronchoscope while pathological results showed no evidence of tumors. Presumed to be pulmonary infection, he was then given piperacillin-tazobactam, moxifloxacin, and empirical anti-tuberculosis treatment successively. His body temperature gradually returned to normal, but the cough still exists. One week later, his cough worsened without fever. Outpatient blood routine examination revealed that white cells count (WBC) 13.1 x 10^9^/*L*, C-reactive protein (CRP) 183.51 mg/L. Re-examination of chest CT showed infectious lesions in both lungs, left hilar shadow increased, and mediastinal lymph nodes slightly enlarged (Figure 1B). For further treatment, he was admitted to the Department of Infectious Diseases of Ningbo First hospital. His initial diagnosis was bacterial pneumonia.

He has not taken any immunosuppressants or glucocorticoids before. He had hypertension for more than 10 years treated by amlodipine and bisoprolol, diabetes mellitus for 3 years treated by insulin with unsatisfactory glycemic control, and eczema for 10 years without therapy. He did not have chronic airway disease although he had smoked for 20 years and quitted smoking for more than 20 years. Three years ago, he had undergone Permanent Cardiac Pacemaker implantation. As a Ningbo native, he had retired from a Metal Instrument Factory where he worked as an installer of metallic parts for more than 20 years. And he hasn't been to the wild recently. He has no prior family history of malignancy, infectious diseases or psychosis. Physical examination showed large eczema-like scars on two legs and found no other positive signs.

After admission, we examined his cellular immune function for lymphocyte subsets analysis (Table 1), immunoglobulins, cytokines, and found no obvious immune deficiency. And his tuberculosis T-SPOT. TB test, human immunodeficiency virus (HIV) test, tumor biomarkers (including carcinoembryonic antigen, carbohydrate associated antigen 199, alpha-fetoprotein, and cancer antigen 125), Antinuclear antibody (ANA) and Antineutrophil autoantibodies (ANCA), serum cryptococcal antigen colloidal gold test, and serological test for syphilis were all negative.

**Table 1:** Lymphocyte subsets analysis.

**Items**	**Results**	**Normal range**
CD3	65.57%	58.4-81.56
CD4	39.72%	31-60
CD8	27.23%	13-41
CD4/CD8	1.5	0.8-4.2
NK	27.09%	14-40
CD19	6.31%	6-25

**Figure 2:**
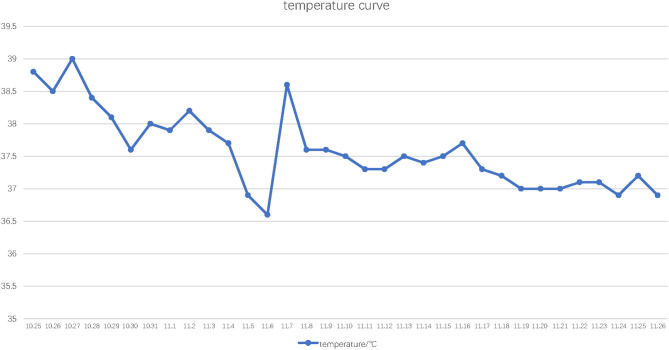
Temperature curve. The horizontal axis indicates the date and the vertical axis indicates highest daily ear temperature.

**Figure 3:**
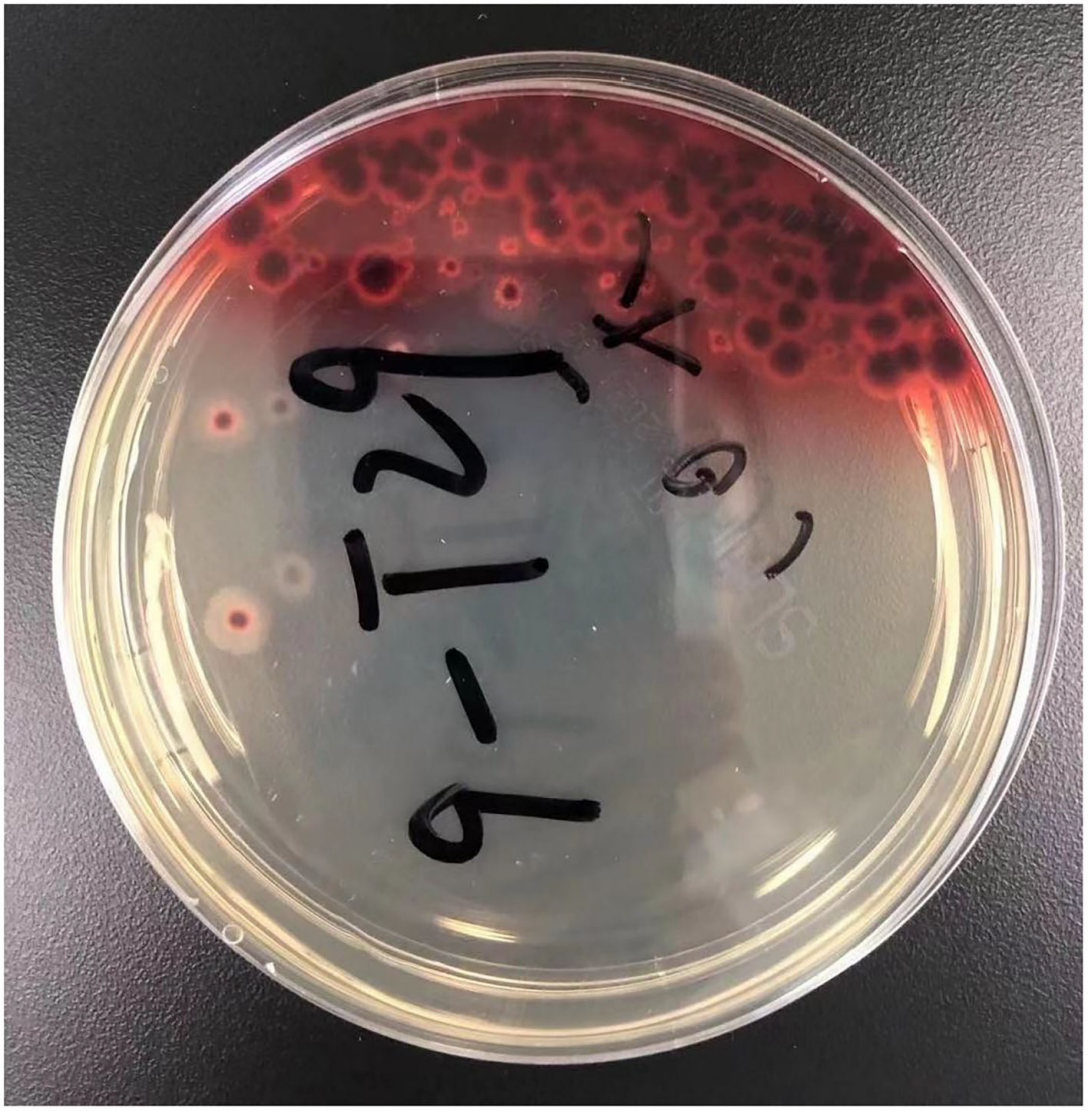
The result of prolonged sputum culture. A diffusible red pigment produced by *Talaromyces marneffei*, diffuses to the surrounding medium.

**Figure 4:**
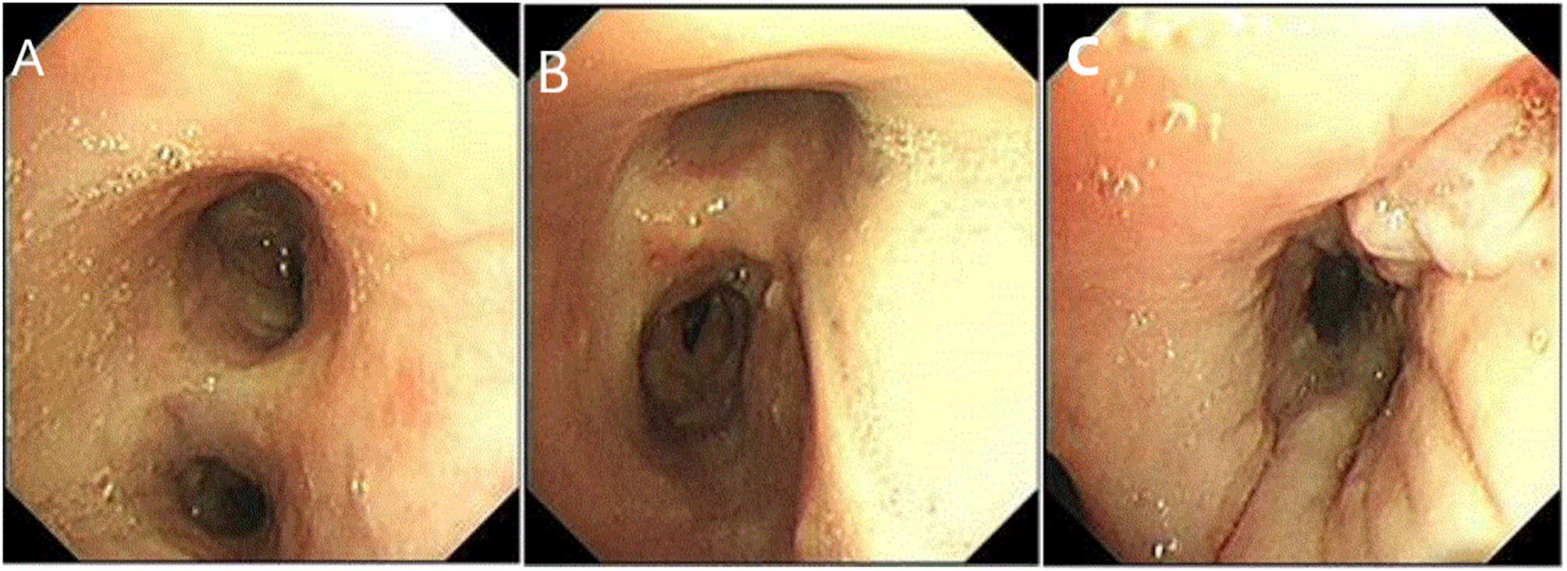
Fiberoptic bronchoscopy. **(A,B)** showed the mucosa of the left and right main bronchus is scattered with nodules. **(C)** Longitudinal change of mucosa in the basal segment of the left lower lobe.

Since he has used broad-spectrum antibiotics with poor efficacy, we performed meropenem, tigecycline consecutively (Table 2). However, the peak temperature did not drop (Figure 2), and the inflammation indexes did not improve (Table 3). During hospitalization, his galactomannan (GM) was positive in serum. Thus, we empirically add caspofungin for antifungal treatment. Meanwhile, we replaced tigecycline with linezolid. Since then, the fever seems to have gradually eased but WBC increased slowly (Figure 3). We ordered another chest CT scan which indicated the lesions of pneumonia were larger than before and pleural fluid appeared (Figure 1C). On November 5, he underwent a fiberoptic bronchoscopic examination (Figure 4) and lavage of the basal segment of the left lower lobe. Bronchoalveolar Lavage fluid (BALF) was tested for mNGS, culture, liquid-based cytology. At the same time, blood sample was sent for mNGS examination.

**Table 2:** The details of therapy strategies.

**Period**	**Therapy**
Oct 25-Oct 28	Meropenem 1 g q8h ivgtt
Oct 28-Nov 1	Tigecycline 50 mg q12h ivgtt
Nov 1-Nov 9	Caspofungin 50 mg qd ivgtt (70mg for first dose)
Nov 2-Nov 9	Linezolid 0.6 g q12h ivgtt
Nov 9-Nov 15	Piperacillin and Tazobactam 4.5 g q8h ivgtt
Nov 9-Nov 26	Voriconazole 200 mg q12h ivgtt
Nov 9-Nov 23	Ganciclovir 250 mg qd ivgtt
Nov 15-Nov 26	Levofloxacin 0.5 g qd ivgtt

**Table 3:** Routine blood test, hsCRP and procalcitonin (PCT).

	**hsCRPmg/L**	WBC*10^∧^9/*L*	**NE%**	RBC*10^∧^12/*L*	**Hb g/L**	PLT *10^∧^9/*L*	**PCT ng/ml**
Oct 25	176.73	13.91	75.1	2.95	81	440	0.42
Oct 27	138.67	14.4	73.7	3.06	80	449	-
Oct 30	173.42	11.64	70.7	3.1	82	527	-
Nov 1	142.18	15.47	71.1	3.2	88	511	-
Nov 3	131.76	17.21	75.1	3.26	88	569	0.33
Nov 5	119.37	18.53	75.2	2.98	86	527	-
Nov 7	80.59	21.89	92.1	3.12	81	478	-
Nov 11	114.77	14.82	78.3	2.89	76	347	-
Nov 14	74.05	11.89	63.9	2.6	70	386	-
Nov 17	63.47	11.47	71.1	2.74	69	550	-
Nov 19	48.72	10.15	73.9	2.72	72	745	-
Nov 22	36.8	10.86	69.9	2.68	72	768	-
Nov 24	31.57	10.85	67.5	2.41	75	761	-
Nov 26	25.41	9.53	66.5	2.54	77	563	-

**Figure 5:**
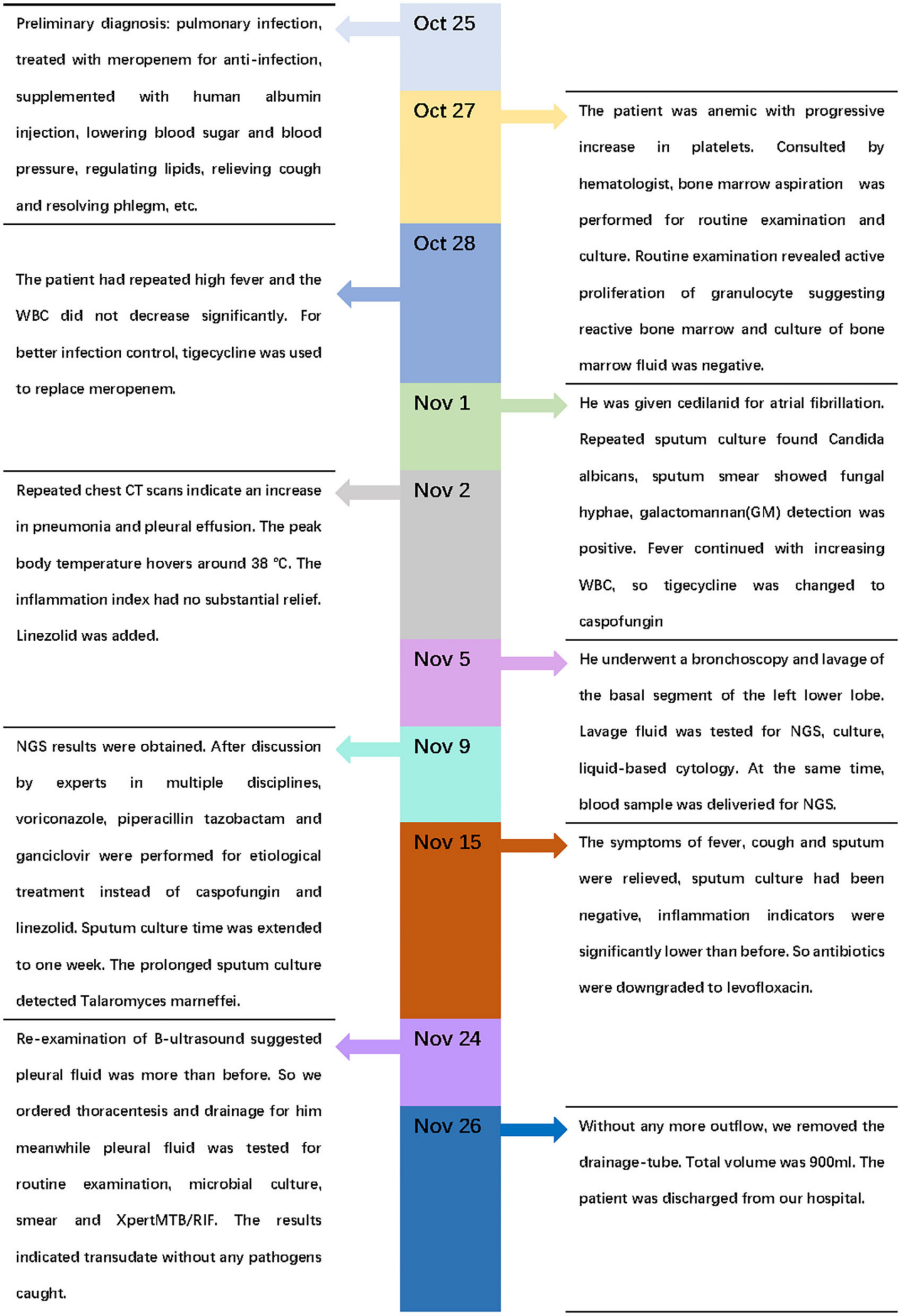
Timeline of the case.

Four days later, we changed antibiotics to intravenous voriconazole and piperacillin-tazobactam since mNGS suggested Penicillium marneffei infection (Table 4). Moreover, we prolonged sputum culture time to one week and the fungus was further validated by culture (Figure 3). After he was prescribed targeted anti-fungal agent of voriconazole 200 mg twice daily, the clinical presentations and laboratory indicators were gradually returning to normal (Table 3). The whole process was concluded (Figure 5).

**Table 4:** Results of mNGS of BALF and blood.

**Sample**	**Type**	**Name**	**Sequence number**	**Confidence**
BALF	G-	*Klebsiella pneumonia*	28	High
BALF	G-	*Pseudomonas aeruginosa*	20	High
BALF	dsDNA	Human betaherpesvirus 5	2	Medium
BALF	Fungi	Talaromycesmarneffei	38	High
BALF	Fungi	*Candida albicans*	5	High
Blood	G-	*Pseudomonas aeruginosa*	66	Medium
Blood	G+	*Staphylococcus aureus*	4	Medium
Blood	dsDNA	Human betaherpesvirus 5	159	High
Blood	dsDNA	Human gammaherpesvirus 4	7	Medium
Blood	Fungi	Talaromycesmarneffei	1	Medium

Prior to follow-up, he carried on taking voriconazole 200 mg twice daily regularly without relapse. On December 9th, his repeated chest CT scan showed that pneumonia was cured and pleural effusion were absorbed obviously (Figure 1D).

## Discussion

As the only thermal dimorphic fungus of Penicillium, *Talaromyces marneffei* grows as yeast *in vivo* at 37°C as pathogenic mode and as mold *in vitro* at 25°C (4). It was originally taken from the hepar of Rhizomys in 1956 while three years later, the first case of human infected by *Talaromyces marneffei* was found derived from the laboratory (1, 2). *Talaromyces marneffei* proliferates in macrophages and spreads through reticuloendothelial system. T cells, especially CD4+ Cells, are vital to remove *Talaromyces marneffei*. In theory, *Talaromyces marneffei* can be cleared in 2–3 weeks in healthy hosts while it may be fatal to immunocompromised patients (1). Thus, it tends to be found on hosts with cellular immune deficiency. The most common hosts are HIV patients followed by other immunocompromised hosts, such as patients who suffer from connective tissue diseases or hematopathy, organ transplant recipients, and long-term users of immunosuppressants. It rarely persists in immunocompetent hosts.

The pathological changes caused by talaromycosis may be divided into three groups as granuloma, reactive necrosis and purulent inflammation (1). The most common clinical symptoms are fever, coughing, anemia, wasting, and characteristic umbilicated skin lesion (5, 6) which are usually nonspecific and of little help to the judgment. Chest CT scan of pulmonary talaromycosis often lacks specificity. As is reported, it can be multiple cavities with uneven thick wall and clear edge, enlarged mediastinal lymph nodes, ground glass opacity, or pleural effusion (1, 7). Thus, without enough suspicion, it is easy to be misdiagnosed as bacterial pneumonia, cancer, tuberculosis, and other fungal infection.

In recent decades, increasing incidents of talaromycosis in healthy hosts are reported (8). The fatality rate of talaromycosis in non-HIV patients is higher than HIV-infected patients which is probably due to delayed diagnoses and treatments (2, 3). Without timely diagnosis and targeted therapy, the mortality rate of talaromycosis can be above 90% (9). We herein reported a case of HIV-negative talaromycosis with pleural effusion from nonendemic region, and without history of recent contact with wildlife. The old man suffered from diabetes with unsatisfactory blood glucose control, cardiac disease and eczema which maybe risk factors of talaromycosis. However, lymphocyte subsets analysis showed his CD3, CD4/CD3, CD8, and CD4/CD8 were all normal in addition to the negative HIV test. Like many other immunocompetent talaromycosis patients, he had been misdiagnosed as bacterial pneumonia and tuberculosis until mNGS cleared away the confusion. Furthermore, prolonged sputum culture verified the pathogen showing bright red surrounding molds which is the unique feature of *Talaromyces marneffei* as it can product a soluble red pigment (3) whose essence maybe monascorubramine encoded by pks3 and other four genes (10). Blood dissemination can be determined from the mNGS of blood. According to literature, disseminated talaromycosis usually has worse prognosis than localized ones since it may be accompanied with multiple organ dysfunction (1). We speculated his anemia maybe related to *Talaromyces marneffei* (11) although it needs further investigation and verification.

Amphotericin B is the first line agent for talaromycosis with HIV suggested by guideline (12). However, severe nephrotoxicity limits the clinical use of amphotericin B. As a broad-spectrum antifungal agent, voriconazole was reported to be effective for talaromycosis (13, 14). Therefore, we chose voriconazole to treat this patient since it is safe, strong and within his economic affordability. The result was satisfactory.

There are some take-away lessons from this case. Since routine culture often cannot cover all bacteria and fungi, timely communications between clinicians and microbiologists is necessary when a particular pathogen is suspected. Microbiologists may then adjust culture methods to improve detection rate of rare pathogens. When traditional anti-infection treatments are not effective and the pathogen is difficult to be detected by routine examinations, mNGS is recommended since it may detect some rare pathogen faster than culture. Then the patient may get the appropriate treatments sooner and have a better prognosis.

Some limitations were discovered in this case. First, in the course of empirical medication, we changed agents frequently which may be influenced by patient's anxiety and high expectations. Second, we lacked suspicion to this rare fungus and initially did not have close communication with microbiology experts which we later rectified. Last, we still don't understand if his thrombocytosis and refractory pleural effusions are related to talaromycosis when his albumin concentration was above 30 g/L.

In conclusion, we should be alert of rare fungi when routine anti-infective therapies were useless even in immunocompetent hosts. As an advanced test method, mNGS may detect pathogens fast and exactly.

## Data Availability Statement

The datasets have been uploaded into EMBL database, accession number PRJEB43434.

## Ethics Statement

Ethical review and approval was not required for the study on human participants in accordance with the local legislation and institutional requirements. The patients/participants provided their written informed consent to participate in this study.

## Author Contributions

JS analyzed data and drafted the manuscript. NY collected the data. GQ revised the manuscript. All authors read and approved the final manuscript.

## Conflict of Interest

The authors declare that the research was conducted in the absence of any commercial or financial relationships that could be construed as a potential conflict of interest.
